# Factors Associated with Extended Hospital Stay and its Impact on Subsequent Short-term Readmission with Tuberculosis Patients

**DOI:** 10.34172/aim.25459

**Published:** 2024-04-29

**Authors:** Jing Cao, Hebin Xie, Zikai Yu, Yu Zhang

**Affiliations:** ^1^The Affiliated Changsha Central Hospital, Hengyang Medical School, University of South China, Changsha 410004, China

**Keywords:** Extended length of stay, Nomograph, Readmission, Tuberculosis

## Abstract

**Background:**

This study aimed to explore the factors associated with extended length of stay (LOSE) for patients with tuberculosis (TB) in China, and construct a nomogram to predict it. In addition, the impact of extended hospital stay on short-term readmission after discharge was assessed.

**Methods:**

A retrospective observational study was conducted at Changsha Central Hospital, from January 2018 to December 2020. Patients (≥18 years who were first admitted to hospital for TB treatment) with non-multidrug-resistant TB were selected using the World Health Organization’s International Classification of Diseases, 10th Revision (ICD-10-CM), and the hospital’s electronic medical record system.

**Results:**

A multivariate logistic regression analysis was used to evaluate the associations between TB and LOSE. The relationship between length of hospital stay and readmission within 31 days after discharge was assessed using a univariate Cox proportional risk model. A total of 14259 patients were included in this study (13629 patients in the development group and 630 in the validation group). The factors associated with extended hospital stays were age, smear positivity, extrapulmonary involvement, surgery, transfer from other medical structures, smoking, chronic liver disease, and drug-induced hepatitis. There was no statistical significance in the 31-day readmission rate of TB between the LOSE and length of stay≤14 days groups (hazards ratio: 0.92, 95% CI: 0.80-1.06, *P*=0.229).

**Conclusion:**

LOSE with TB was influenced by several patient-level factors, which were combined to construct a nomograph. The established nomograph can help hospital administrator and clinicians to identify patients with TB requiring extended hospital stays, and more efficiently plan for treatment programs and resource needs.

## Introduction

 Improving hospital quality and reducing costs remain some of the most critical issues faced by patients, providers, and managers worldwide. Length of stay (LOS) measures the rate of utilization of hospital beds and the efficiency of the admissions. Therefore, LOS is considered to be an indicator of hospital service efficiency and effectiveness, as well as hospital management effectiveness.^1,2^ In addition, LOS is essential in determining total hospitalization costs.^3^ According to a cross-sectional study,^4^ each extended hospitalization day for a single patient increased costs by 1022.853 ETB (Ethiopian birr). Some studies also suggest that a longer LOS may lead to increased risk of readmission and iatrogenic complications.^5^

 Tuberculosis (TB) is a chronic respiratory tract infectious disease that seriously endangers human health. The causative pathogenic bacteria, *Mycobacterium tuberculosis*, can be transmitted through the air following the patient’s bacterial discharge. Although TB most commonly affects the lungs, it can also affect other organs (extrapulmonary tuberculosis [EPTB]).^6^ China has the second-largest number of TB cases globally, accounting for approximately 9% of the global TB incidence.^7^ Although the Chinese government provides free diagnostic measures and anti-TB drugs for patients with TB, it is still common for the patients to face financial burden resulting from hospitalization costs. However, studies on the LOS in hospital for patients with TB are few, with none in China or Asia.

 This study aimed to explore the factors associated with extended hospital stay for patients with TB in China, and construct a nomogram to predict the extended length of stay (LOSE) in TB; In addition, the impact of extended hospital stay on short-term readmission after discharge was also assessed.

## Materials and Methods

###  Data Sources and Study Population

 We conducted a retrospective observational study using the Changsha Central Hospital database between January 1, 2018, and December 31, 2020, and collected the patients’ basic information and clinical data. Identification of patients with TB was based on the World Health Organization’s International Classification of Diseases, 10^th^ Revision (ICD-10-CM) codes, and the principal diagnoses recorded in the hospital electronic medical system. We studied non-mDR-TB (including TB and EPTB) patients aged ≥ 18 years who were first admitted to the hospital for TB treatment. W excluded cases of multidrug-resistant TB, drug-induced liver injury (DILI) not caused by anti-tuberculous treatment, TB patients hospitalized for other complications, pregnant or lactating women, co-infection with HIV, liver cirrhosis, chronic renal insufficiency, malignant tumors, and those who died during hospitalization.

###  Extended Length of Stay, Readmission, and Severe Tuberculosis

 LOSE was defined as a hospital stay greater than 75th percentile for the entire study population ( > 14 days), based on previous reports and combined with the actual situation in China.^8-10^ Readmission was defined as patient’s readmission to the hospital within 31 days after discharge (due to TB-related illness). Severe TB is diagnosed if one the following conditions is met: (1) The lesion range is more than 3 lobes, and the symptoms of TB poisoning are severe (the body temperature is less than 39 ℃); (2) TB of two or more organs (except TB with tuberculous pleuritis).^11^

###  Statistical Analysis 

 The software environment R (R version 3.3.2) and the STATA software version 13.1 (Stata Corp., College Station, TX) were used for statistical analysis. The follow-up period spanned the first day to 31 days after the patient was discharged from the hospital, and the endpoint was readmission (due to TB) within 31 days of discharge. The follow-up survey was conducted using a WeChat or telephone review, and the proportion of censored observations was 1.17% (The reason for censoring was that the patient died or lost contact during follow-up). The association between length of hospital stay and readmission within 31 days after discharge was assessed using univariate Cox proportional risk models. The associations between variables and the risk of LOSE were evaluated using univariate logistic analysis. Multivariable analysis was performed using logistic regression (with enter methods), including all variables with a *P* value < 0.1. We performed the Box-Tidwell test to examine the linearity assumption for quantitative predictors and the dependent variable in the logistic regression model. A nomogram prediction model was developed in the R software, and the discriminative ability of the nomogram was evaluated by the area under the receiver operating characteristic curve (ROC). Calibration of the nomogram was assessed by plotting the observed outcome probabilities and the probabilities predicted by the logistic model. A two-tailed *P* value < 0.05 was regarded as statistically significant.

## Results

 Totally, 13 629 patients were included in the development groups, and 630 patients were included in validation groups. For the development cohort, more than half (66.27%) of the patients were males, and the median age was 54 years; LOS ranged from 1 to 336 days, and the median was 9 days. For the validation cohort, 63.78% of the patients were males, and the median age was 52 years; LOS ranged from 3 to 312 days, and the median was 8 days.

 Based on the results of simple logistic regression in the development cohort, age, marital status, sputum smear, form of TB, surgery, form of hospitalization, severe TB, smoking, coronary heart disease, chronic liver disease, and drug-induced hepatitis achieved the statistical significance of *P* < 0.1 and were included as potential covariates (Table 1). During our study, 810 patients (5.94%) were readmitted because of TB within 31 days after the first discharge. The LOSE group had a median follow-up of 26 days (IQR, 17–29 days), and the LOS ≤ 14 days group had a median follow-up of 27 days (IQR, 18–30days). There was no statistical significance in the 31-day readmission rate of TB between the LOSE and LOS ≤ 14 days groups, as shown by univariate cox analysis (hazards ratio: 0.92,95% confidence interval:0.80–1.06, *P* = 0.229).

**Table 1:** Factors Associated with Extended Hospital Stay in Patients with Tuberculosis

**Variables**		**LOS≤14 days(n=10312)**	**LOS>14 days (n=3317)**	**Univariate Analysis**		**Multiple Analysis**	
				**OR (95% CI)**	***P *** **Value**	**OR (95% CI)**	***P *** **Value**
Gender, No. (%)	Male	6857 (66.5)	2176 (65.6)	1.04 (0.96-1.13)	0.344		
	Female	3455 (33.5)	1141 (34.4)	1			
Age group		51.28 ± 18.04	52.18 ± 18.74	1.02 (1.01-1.05)	0.014	1.05 (1.03-1.10)	< 0.001
Marital status, No. (%)	Single	1529 (14.83)	533 (16.07)	1		1	
	Married	8297 (80.46)	2613 (78.78)	0.90 (0.81-1.01)	0.065	0.76 (0.67-1.03)	0.109
	Divorced	142 (1.38)	51 (1.54)	1.03 (0.74-1.44)	0.861	0.74 (0.52-1.06)	0.100
	Widowed	344 (3.34)	120 (3.62)	1.00 (0.8-1.26)	0.995	0.75 (0.57-1.19)	0.320
Payment method, No. (%)	Urban employee basic medical insurance	2376 (23.04)	778 (23.45)	0.97 (0.79-1.18)	0.730		
	Urban resident medical insurance	2375 (23.03)	711 (21.44)	0.88 (0.72-1.08)	0.221		
	New rural cooperative medical insurance	4483 (43.47)	1490 (44.92)	0.98 (0.81-1.19)	0.836		
	Self-paying	615 (5.96)	181 (5.46)	0.87 (0.68-1.11)	0.258		
	Other commercial insurance	463 (4.49)	157 (4.73)	1			
Residency, No. (%)	Rural	6388 (61.95)	2026 (61.08)	1	0.371		
	Urban	3924 (38.05)	1291 (38.92)	1.04 (0.96-1.12)			
Sputum smear, No. (%)	Smear negative	8142 (78.96)	2395 (72.2)	1	< 0.001	1	< 0.001
	Smear positive	2170 (21.04)	922 (27.8)	1.44(1.32-1.58)		2.03 (1.84-2.25)	
Form of TB, No. (%)	Intrapulmonary	9217 (89.38)	2387 (71.96)	1	< 0.001	1	< 0.001
	Extrapulmonary	1095 (10.62)	930 (28.04)	3.28 (2.97-3.62)		4.18 (3.75-4.66)	
Surgery, No. (%)	No	7238 (70.19)	1697 (51.16)	1	< 0.001	1	< 0.001
	Yes	3074 (29.81)	1620 (48.84)	2.25 (2.07-2.44)		2.16 (1.99-2.35)	
Form of hospitalization, No. (%)	Emergency room	797 (7.73)	301 (9.07)	1	< 0.001	1	
	Out-patient clinic	9437 (91.51)	2959 (89.21)	0.83 (0.73-0.95)	0.009	0.98(0.84-1.15)	0.825
	Transfer from other medical structures	78 (0.76)	57 (1.72)	1.94 (1.34-2.79)	< 0.001	1.84(1.24-2.73)	0.003
Severe TB, No. (%)	No	8637 (83.76)	2691 (81.13)	1	< 0.001	1	0.189
	Yes	1675 (16.24)	626 (18.87)	1.20 (1.08-1.33)		1.08(0.96-1.20)	
Smoking, No. (%)	No	9685 (93.92)	2892 (87.19)	1	< 0.001	1	< 0.001
	Yes	627 (6.08)	425 (12.81)	2.27 (1.99-2.59)		3.03(2.64-3.48)	
Drinking, No. (%)	No	9692 (93.99)	3143(94.75)	1	0.101		
	Yes	620 (6.01)	174 (5.25)	0.87 (0.73-1.03)			
Hypertension, No. (%)	No	8731 (84.67)	2828(85.26)	1	0.411		
	Yes	1581 (15.33)	489 (14.74)	0.95 (0.86-1.07)			
Coronary heart disease, No. (%)	No	9519 (92.31)	3094 (93.28)	1	0.065	1	0.189
	Yes	793 (7.69)	223 (6.72)	0.87 (0.74-1.01)		0.84 (0.71-1.01)	
COPD, No. (%)	No	9520 (92.32)	3089 (93.13)	1	0.125		
	Yes	792 (7.68)	228 (6.87)	0.89 (0.76-1.03)			
Chronic liver disease, No. (%)	No	7627 (73.96)	2232 (67.29)	1	< 0.001	1	< 0.001
	Yes	2685 (26.04)	1085 (32.71)	1.38 (1.27-1.50)		1.34 (1.22-1.47)	
Complications, No. (%)	No	10299 (99.87)	3312 (99.85)	1	0.734		
	Yes	13 (0.13)	5 (0.15)	1.20 (0.43-3.36)			
Drug- induced hepatitis, No. (%)	No	10227(99.18)	3215(96.92)	1	< 0.001	1	< 0.001
	Yes	85 (0.82)	102 (3.08)	3.82 (2.85-5.10)		3.44 (2.50-4.73)	
Comorbidities ≥ 3, No. (%)	No	9047 (87.73)	2935 (88.48)	1	0.249		
	Yes	1265 (12.27)	382 (11.52)	0.93 (0.82-1.05)			
Clinical pathway, No. (%)	Complete	8371 (81.18)	2736 (82.48)	1			
	Not entered	1863 (18.07)	563 (16.97)	0.92 (0.83-1.03)	0.138		
	Quit	78 (0.76)	18 (0.54)	0.71 (0.42-1.18)	0.185		

 We performed the Box-Tidwell test to assess the linearity assumption for age and the dependent variable in the logistic regression model. We found that the interaction of age and In (age) had no statistical significance (*P* = 0.098), which means that the quantitative predictor in our study met the linearity assumption. The final multivariable analysis suggested that sociodemographic variables age, form of hospitalization (transfer from other medical structures), clinical features (sputum smear, form of TB, surgery, drug-induced hepatitis), and comorbidities (chronic liver disease) were associated with higher odds of extended hospital stay (all *P*< 0.05) (Table 1).

**Figure 1:**
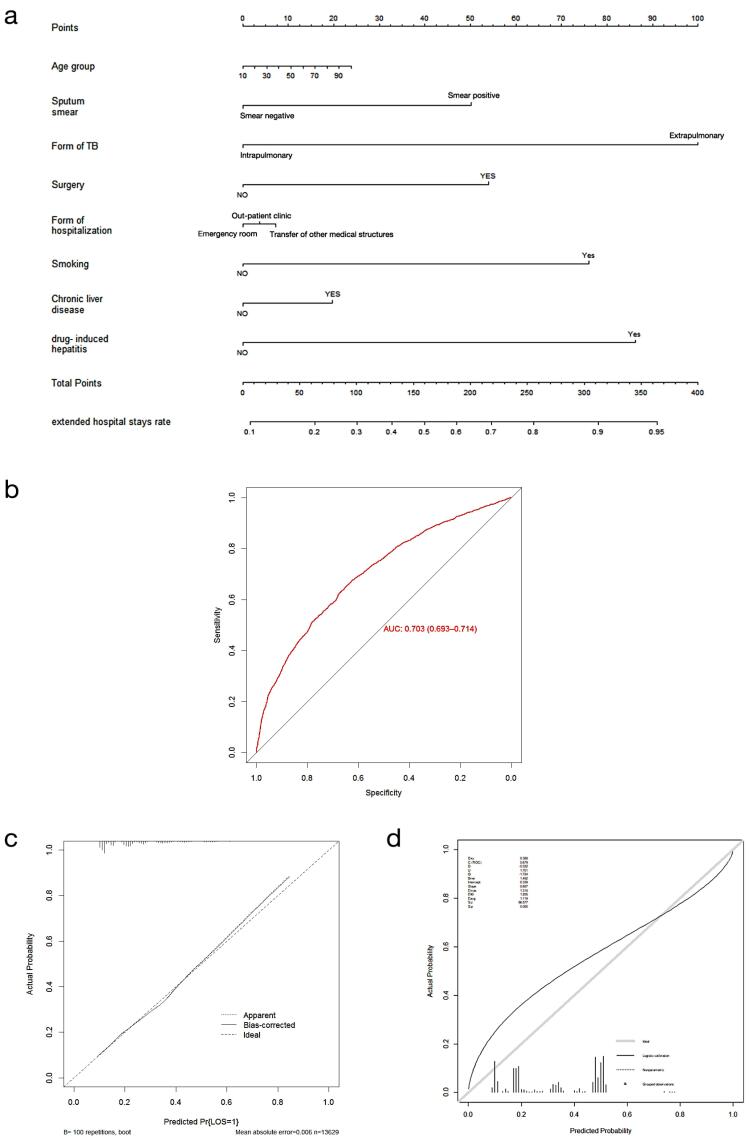
(a) A constructed nomogram for prediction extended hospital stays of patients with tuberculosis. (b) Receiver operating characteristics (ROC) curve figure of the nomogram. (c) Development cohort calibration curves. (d) Validation cohort calibration curves

 Independent risk factors were constructed into a nomogram model (Figure 1a), in which different variables were quantified into specific scores. After the internal self-sampling (bootstrap) test, the calibration curve of the model was obtained with an average absolute error of 0.006, which suggested that the predicted probability was close to the actual situation and that the model showed good consistency (Figure 1b). Model discrimination was deemed excellent with an area under the ROC curve (95% CI) of 0.703 (0.693–0.714); the corresponding sensitivity was 0.658 (0.642–0.674), and the specificity was 0.643 (0.634–0.652). The calibration plots performed well in the development and validation cohort (Figures 1c and 1d).

## Discussion

 In this study, the median length of hospital stay was 9 (interquartile range, 7 to 14) days, similar to results from a study in Spain (11.3 ± 7.0 days),^12^ and lower than a study from the United States (19.5 days) that included pulmonary and EPTB patients.^13^ Of the 810 patients, 5.94% were readmitted because of TB within 31 days after discharge.

 In China, the incidence of TB in the elderly is significantly higher than that in the non-elderly.^14^ As expected, patients older than 60 years were significantly associated with extended hospitalization. In addition, reduced immune function in older adults may lead to increased susceptibility to TB and resurgence of underlying TB infection.^15^ Research has also shownthat lung inflammation increases with age at the individual level,^16^ increasing the risk of extended hospitalization in the elderly. Globally, nearly 20% of TB cases are caused by smoking.^17^ Smoking (active or passive) is an independent risk factor for TB infection and severe disease. According to pathophysiological studies,^18^ the plasma level of rifampicin in smokers is lower than non-smokers. Smoking can affect the efficacy of drugs and cause longer hospital stays.

 Our study shows that 14.86% of TB admissions were EPTB, including meningeal and central nervous system (10.52%), bones and joints (30.32%), urinary and reproductive systems (7.21%), lymphatic (25.19%), miliary TB (13.28%), and other organs (13.48%). Because of the low bacterial load of non-respiratory specimens and the difficulty of collecting samples from deep tissues, diagnosis of EPTB is more complex than pulmonary TB.^19^ In addition, most patients with EPTB need surgical treatment. Consistent with other studies,^13^ EPTB has longer hospital stay, especially for EPTB requiring surgery, compared with TB.

 Isoniazid (INH), rifampicin, pyrazinamide, and ethambutol are the first-line anti-TB drugs, but they can cause hepatotoxicity. Zhao et al^20^ reported that the average time from initiation of anti-TB treatment to progression to DILI is about 24 days. A retrospective study in South Korea reported that HCV infection and HBV + HCV co-infection were independent risk factors for DILI.^21^ Even if the laboratory liver function test of patients with chronic liver disease is normal, there is a certain degree of liver damage, liver pharmacokinetics change, and reduction in drug metabolism enzyme activity, resulting in high drug concentration and prolonged residence time in the liver, and an increased risk of DILI.^22^ The results of this study showed that drug-induced hepatitis and chronic liver disease were risk factors for prolonged hospital stay in patients with TB.

 The hierarchical medical system is an important measure to solve the problem of unbalanced allocation of medical resources and patient flow to general hospitals in China.^23^ The medical treatment alliance refers to regional medical association integrating different medical resources in the same region; it is an effective way to achieve graded diagnosis and treatment. As a tertiary hospital of the Changsha medical union, our hospital accepts TB referral patients from the community and the first- and second-level hospitals. Most of the patients transferred to our hospital presented with complex miscellaneous diseases or emergency and severe cases that lower-level hospitals cannot handle. The treatment and diagnosis of such cases are relatively difficult, with delays in referrals and missed optimal treatment times leading to prolonged hospital stays.

 In recent years, 31-day readmission has been recognized as an important indicator of hospital management and medical technology.^24^ Our study found that the main reasons for readmission within 31 days were fever, dyspnea, pneumothorax, and hemoptysis. Studies in Japan revealed that a shorter LOS was associated with increased rates of 30-day chronic heart failure readmission.^25^ On the other hand, the results of a retrospective study showed that prolonged LOS was associated with a higher risk of readmission in adult neurosurgery patients.^26^ Reynolds et al^27^ reported no significant association between short LOS and early readmission. Similarly, our univariate COX analysis showed no association between the length of TB hospital stay and 31-day readmission.

 Our study has certain limitations. This is a retrospective, single-center study. We further expanded the included sample size based on the calculated effective sample size. However, other factors that might influence the length of hospitalization, such as economic status, nutritional status, educational background, and family support, were not collected and data availability was limited. So, prospective, multi-center studies should be carried out in the future.

 In conclusion, we found that LOSE was not associated with the 31-day readmission of TB. Instead, LOSE of TB was influenced by several patient-level factors including demographic characteristics and behavioral habits (age, smoking), preadmission comorbidities (chronic liver disease), form of hospitalization (transfer from other medical structures), clinical features, and treatment methods (sputum smear, surgery). However, adverse drug reactions and forms of TB were the most important causes of prolonged hospital stay. These factors were combined to construct a nomogram to help hospital administrators and clinicians to identify patients with TB requiring extended hospital stays, and more efficiently plan for treatment programs and resource needs.

## Conclusion

 In conclusion, we found that LOSE was not associated with the 31-day readmission of TB. Instead, LOSE of TB was influenced by several patient-level factors including demographic characteristics and behavioral habits (age, smoking), preadmission comorbidities (chronic liver disease), form of hospitalization (transfer from other medical structures), clinical features, and treatment methods (sputum smear, surgery). However, adverse drug reactions and forms of TB were the most important causes of prolonged hospital stay. These factors were combined to construct a nomogram to help hospital administrators and clinicians to identify patients with TB requiring extended hospital stays, and more efficiently plan for treatment programs and resource needs.
